# Flow cell for *operando* X-ray photon-in-photon-out studies on photo-electrochemical thin film devices

**DOI:** 10.12688/openreseurope.14433.1

**Published:** 2022-06-07

**Authors:** Philipp Jäker, Dino Aegerter, Till Kyburz, Roman Städler, Rea Fonjallaz, Blanka Detlefs, Dorota Koziej

**Affiliations:** 1 Department of Materials, Laboratory for Multifunctional Materials, ETH Zürich, Zurich, Vladimir-Prelog-Weg 5, 8093, Switzerland; 2 Institutes of Nanostructure and Solid State Physics, Center for Hybrid Nanostructures, University of Hamburg, Hamburg, Luruper Chaussee 149, 22607, Germany; 3 European Synchrotron Radiation Facility, Grenoble, 71 avenue des Martyrs, CS 40220, 38043, France

**Keywords:** Operando, operando instrumentation, measurement cell, HERFD-XAS, X-ray absorption spectroscopy, photoelectrochemical, photoelectrochemical water splitting, thin films, metal oxides

## Abstract

**Background:** Photo-electro-chemical (PEC) water splitting represents a promising technology towards an artificial photosynthetic device but many fundamental electronic processes, which govern long-term stability and energetics, are not yet fully understood. X-ray absorption spectroscopy (XAS), and particularly its high energy resolution fluorescence-detected (HERFD) mode, emerges as a powerful tool to study photo-excited charge carrier behavior under operating conditions. The established thin film device architecture of PEC cells provides a well-defined measurement geometry, but it puts many constraints on conducting *operando* XAS experiments. It remains a challenge to establish a standardized thin film exchange procedure and concurrently record high-quality photoelectrochemical and X‑ray absorption spectroscopy data that is unperturbed by bubble formation. Here we address and overcome these instrumental limitations for photoelectrochemical *operando *HERFD-XAS.

**Methods:** We constructed a novel *operando* photo-electro-chemical cell by computer numerical control milling, guided by the materials’ X‑ray and visible light absorption properties to optimize signal detection. To test the cell’s functionality, semiconducting thin film photoelectrodes have been fabricated *via *solution deposition and their photoelectrochemical responses under simulated solar light were studied using a commercial potentiostat in a three-electrode configuration during HERFD-XAS experiments at a synchrotron.

**Results:** We demonstrate the cell’s capabilities to measure and control potentiostatically and in open‑circuit, to detect X‑ray signals unperturbed by bubbles and to fluently exchange different thin film samples by collecting high-resolution Fe K-edge spectra of hematite ( *α* -Fe _2_O _3_) and ferrite thin film ( *M*Fe _2_O _4_, *M*= Zn, Ni) photoelectrodes during water oxidation.

**Conclusions:** Our cell establishes a measurement routine that will provide experimental access of photo-electro-chemical *operando *HERFD-XAS experiments to a broader scientific community, particularly due to the ease of sample exchange. We believe to enable a broad range of experiments which acquired fundamental insights will spur further photoelectrochemical research and commercialization of water splitting technologies

## Plain language summary

We are confronted with the profound societal challenge to completely decarbonize our energy infrastructure. More than the eventual depletion of fossil fuels it is due to the imminent danger of climate change. To stop greenhouse gas emissions, mainly carbon dioxide, means either to establish a closed-loop carbon cycle or to end producing CO _2_ at all. However, recapturing previously released CO _2_ is the least economically and technologically feasible approach. Thus, stopping CO _2_ emissions practically requires to transition from thermal power plants and combustion engines to wind and solar power as well as electric engines. But fossil fuels are extremely dense energy carriers and cannot be always replaced by batteries. For stationary energy storage over long periods and for long range energy extensive transportation such as planes and ships other chemical fuels such as hydrogen will be needed. One approach to generate H _2_ is *via* photoelectrochemical (PEC) water splitting which basically combines a solar cell that converts sunlight into electricity and an electrolyzer that further converts electricity into chemical energy within a signal device. Scientists develop new *operando* measurement techniques for real-time monitoring under real world conditions to bridge the divide between controlled laboratory conditions and complex real world environments. We designed a new measurement cell that allows to look into photoelectrochemical processes using X-ray spectroscopic techniques. We overcame the challenge to accommodate all features necessary to enable smooth photoelectrochemical operation and to extract new information from X-ray measurements. Moreover, we created a very user friendly cell where sample exchange is easy and fast. We believe this will greatly increase adoption of operando X-ray based techniques to study photoelectrochemical water splitting, provide valuable insights to optimize this technology and contribute to solving an important societal issue.

## Introduction

Photo- and electrochemical thin film devices are ubiquitous in current energy conversion technologies such as solar cells or electrolyzer. Their principal geometry enables precise control and determination of surface area, thickness and optical path lengths, which are fundamental to determine related physical properties of materials like the absorption coefficient, conductivity and device performance metrics such as current density. Photo-electro-chemical (PEC) water splitting represents a promising thin film based application integrating photovoltaic and electrosynthetic functions within a single device to produce hydrogen as energy storage medium ^1, 2^. Overarching research goals on the path towards commercialization are the realization of conversion efficiencies competitive with solar thermal water splitting or wind and solar power driven electrolyzers and long term device stability (~ 10 - 20 years) ^3– 5^. Due to the topical complexity, a common research strategy relies on explorative and iterative materials engineering ^6– 8^. We observe continously raising energy conversion efficiencies, but underlying photoelectrochemical processes still remain unexplored and thus we are lacking a more fundamental understanding of this technology ^9^.

In PEC devices, semiconductor liquid electrolyte interfaces are modelled analogous to Mott-Schottky contacts, for which physical parameters are not assumed to be modified during operation ^10^. However, based on recent results for BiVO _4_, an n-type semiconductor photoanode, photocorrosive processes alter the material’s chemical and morphological state during operation ^11, 12^. X-ray absorption spectroscopy (XAS) is particularly appealing to study materials and devices under working conditions due to its high penetration depth and chemical selectivity ^13, 14^. Moreover, in HERFD (high energy resolution fluorescence-detected)-XAS, the energy resolution is enhanced by wavelength dispersive detection of the X-ray fluorescence, giving access to subtle spectral feature variations such as observed in CO _2_ sensing ^15^. *Operando* experiments provide new insights such as an electrochemically induced surface layer ^16^ or light excited charge transfer ^17^ and are becoming common to analyze electrochemical (electrocatalysis, batteries) ^18, 19^ and photochemical processes ^20^. In these areas, the fundamental physical interaction is either electrical or optical and the device only needs to provide electrical connection or light transparency but not both. Device operating constraints for purely electrochemical or photochemical *operando* cells are lowered and thus simplify the *operando* cell design. However, to study photo-electro-chemical processes both qualities have to be ensured simultaneously, increasing the complexity of the cell design.

**Figure 1.**
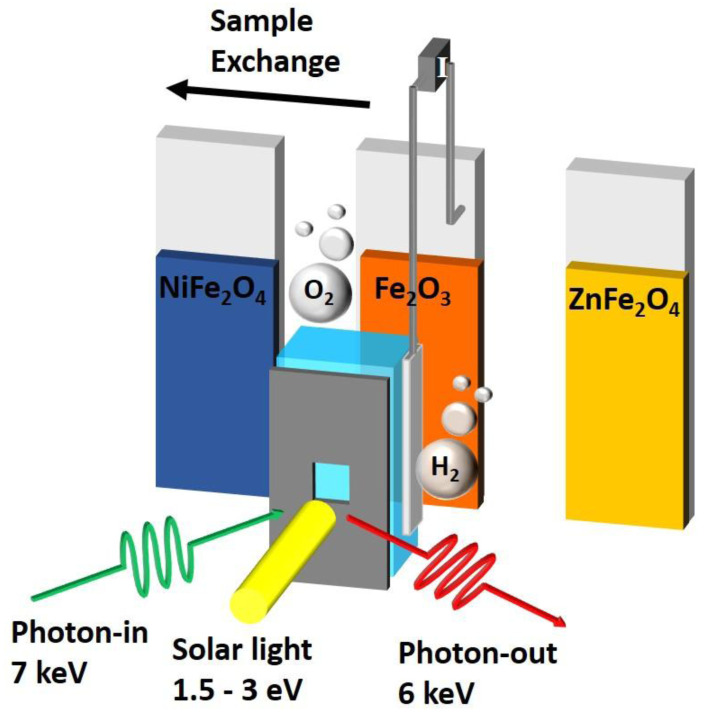
Illustrating the key operando cell characteristics of a fast thin film sample exchange, concurrent X ray excitation (green) and solar excitation (yellow) while measuring the X ray fluorescence (red) and electrochemical (grey electrodes and wires) response.

The few existing examples of *operando* XAS photo-electro-chemical studies interpret their findings as photoexcited charge transfer, either between atoms of two different materials as in the case of Nb:SrTiO _3_/MnO _x_^21^ and Fe _2_O _3_/IrO _x_^22^ or within the same material as for Fe _2_O _3_^23^. In semiconductor photoelectrochemistry the challenge is to disentangle effects caused by the electrical bias and light absorption. Photoinduced phenomena are often studied using pump-probe techniques utilizing coherent monochromatic laser light ^24^. However, lasers high irradiance leads to numerous excited states that are only probed in narrow spectral regions and might not be representative of the exitations induced by solar light in a broad spectral region. Here, we are interested in studying processes under realistic conditions utilitzing standard PEC devices architecture under full solar spectral illumination. To this end, the cell requires three electrodes to record electro-chemical current voltage (I-V) signals and shall be designed to minimize solar light and X-ray attenuation. Current cell versions are limited by electrochemical noise ^21^ or uncessarily high X-ray beam attenuation ^25^. A further challenge is a complicated and irreversible sample exchange mechanism. For instance, depositing samples directly onto the cell`s window material, such as Si _3_N _4_, transforms the costly part into a non reusable one ^23^. We address the above mentioned shortcomings by realizing an *operando* cell that enables us to record high-quality X-ray absorption spectra and to control current voltage responses with low electrochemical noise. Switching thin film materials for sequential *operando* measurements is achieved in a very straightforward manner and enables reproducible experiments on a series of samples ( Figure 1).

## Methods

### Chemicals

The full list of chemicals used in this research are as follows:Acetonitrile (ACN), Sigma-Aldrich, 99.8%; Benzyl alcohol, Sigma-Aldrich, 99.8% anhydrous; CuSO _4_, Sigma-Aldrich, anhydrous, powder, ≥99.99% trace metals basis; Ethanol (EtOH), Sigma-Aldrich, standard for GC; Diethylether (Et _2_O), Sigma-Aldrich, Laboratory Reagent, ≥99.5% (GC); Isopropanol (IPA), Alfa Aesar, ACS, ≥99.5%. 2- 2-(2-methoxyethoxy)ethoxy acetic acid (MEEAA), Sigma-Aldrich, technical grade; NaOH, Sigma-Aldrich, reagent grade, ≥98%, pellets (anhydrous); NH _4_OH, Sigma-Aldrich, puriss. p.a., reag. ISO, reag. Ph. Eur., ~25% NH3 basis; Fe(acac) _3_, Sigma-Aldrich, 97%; Zn(OAc) _2_, Sigma-Aldrich, 99.99% trace metal basis; Ni(OAc) _2_×4 H _2_O, Sigma-Aldrich, 99.99% trace metal basis. All chemicals were used without further purification.

### Nanoparticle synthesis

Ferrite oxide nanoparticles ( *M*Fe _2_O _4_, *M*= Zn, Ni, Fe, Co, Mn) are synthesized using a slightly modified benzyl alcohol route ^26, 27^. All particles were prepared in 10 mL benzyl alcohol poured into a 25 ml round-bottom glass flask. To obtain ZnFe _2_O _4_ nanocrystals, 1 mmol (354 mg) of Fe(acac) _3_ and 0.5 mmol (90 mg) of Zn(OAc) _2_ were used as precursors. NiFe _2_O _4_ nanoparticles (NPs) are produced from 1 mmol (354 mg) of Fe(acac) _3_ and 0.5 mmol (125 mg) of Ni(OAc) _2_×4 H _2_O. Use of only 1 mmol (354 mg) of Fe(acac) yield Fe _3_O _4 _NPs that were precursors to form Fe _2_O _3_ NPs upon calcination. All chemicals are stored inside a glovebox under Argon atmosphere. To dissolve precursors in benzyl alcohol the flask is kept at 100 °C for two hours in an oil bath and then transferred to a preheated 220 °C hot oil bath to avoid the formation of undesired zinc oxides phase. The flask is cooled down to room temperature (RT) and its content is completely poured into a 50 ml centrifuge tube. The flask is rinsed with few mL of Et _2_O and added to the centrifuge tube. The Et _2_O induces precipitation of the nanoparticles. Centrifugation for 30 min at 4000 rpm separates the nanoparticles from the liquid. The supernatant is discarded and the tube is refilled with 30 ml of a 1:1 solution of EtOH and Et _2_O. The sedimented solid residue is whirled up and the tube is centrifuged again for 30 min at 4000 rpm. This process is repeated twice. The solid residue is dried at 60 °C for two hours and finely ground.

### Photoelectrode preparation

Photoanode thin films are fabricated from preformed ferrite oxide ( *M*Fe _2_O _4_, *M*=Zn, Ni, Fe, Co, Mn) nanoparticles by spin-coating on fluorine-doped tin oxide (FTO) covered glass substrates (1.5 cm × 3.5 cm, 7 Ω/sq, Solaronix). Prior to use, each FTO substrate is cleaned according to the following procedure. The substrate is consecutively placed into a solution of soap (Migros) water, absolute EtOH and deionised water while being sonicated for 15 min. Afterwards, it is immersed into an IPA solution and dried in a nitrogen stream. The spin-coating solution is prepared by dispersing 60 mg of ferrite nanoparticles in a homogeneous mixture of 940 μl ACN, 40 μl deionised water and 20 μl MEEAA which functions as stabilizer ^27– 29^. After sonicating for 30 min larger agglomerates are removed by filtration to increase the dispersion’s (ink) stability with a Chromafil Xtra PTFE-45/25 Syringe Filter from Macherey-Nagel of 450 nm in pore size. Next, 60 µL ink is spin-coated onto FTO for thin film preparation. We employ a WS-650-23NPP Spin Coater from Laurell Technologies to spin the substrate for 30 s at 4000 rpm in nitrogen atmosphere. Immediately after spin-coating the substrates are dried at 300 °C for 10 min using a pre-heated MR 3004 Safety Hotplate from Heidolph and are let to cool down to RT. Next, the substrates were covered by a ceramic bowl and are heated to 700 °C at a rate of 10 °C min ^-1^ for 2 hours in a RHF16/3/3216P1 High-Temperature Box Furnace from Carbolite. The temperature treatment transforms Fe _3_O _4_ into Fe _2_O _3_ nanoparticles whereas ZnFe _2_O _4_ and NiFe _2_O _4_ preserve their crystalline phase. Grazing Incidence X-ray diffraction (GIXRD) confirms crystallinity and phase purity as shown in Figure S5 (see *Extended data* ^30^). We determine film thickness from cross-sectional scanning electron microscopy images (SEM) to about 50–80 nm exemplary shown for ZnFe _2_O _4_ in Figure S3 (see *Extended data* ^30^).

### Gracing-incidence X-ray diffraction (GIXRD)

GIXRD experiments of all thin films were conducted with a PANalytical X’Pert Pro producing Cu-Kα _1,2_ radiation that is parallelized using a parabolic W/Si X-ray mirror. The incident beam size is reduced by a 1/32° slit and its intensity is lowered using a 0.125 mm thick Ni-based programmable beam attenuator. The reflected beam passes a 0.18° parallel plate collimator and is detected on a Xe-proportionality detector. First, an X-ray reflectivity scan in Omega-2Theta geometry was performed to determine the critical angle of total reflection. Afterwards, a slightly higher fixed incident beam angle at around 0.7° was chosen for GIXRD measurements in 2theta geometry. Raw data and the explicit data treatment (normalization and vertical translation) using OriginPro 2017 b9.4.0.220 are accessible from *Underlying data* ^30^. Freely available software alternatives for data treatment and plotting are SciDavis or LabPlot.

### Scanning electron microscopy (SEM)

SEM cross-sectional images were taken on a Zeiss Leo 1530 equipped with an in-lens detector. Electrons were accelerated with 3 kV and the beam was scanned across the thin film at a working distance of 4.8 mm. ImageJ ^31, 32^ was used to enhance contrast and brightness of the whole image and to crop out the relevant section.

### Photographs

Optical images of the *operando* cell and the thin films were taken with a Panasonic DMC FZ200 under variable magnification and exposure time. ImageJ 1.51j8 was used to enhance contrast and brightness of the whole image and to crop out the relevant section.

### Crystal structure visualization

The VESTA 3.4.0 program ^33^ was used to visualize the crystal structures. Crystallographic Information Files (CIFs) of Fe _2_O _3_^34^ (ICSD collection code 40142) and ZnFe _2_O _4_^35^ (ICSD collection code 75097) were obtained from the Inorganic Crystal Structure Database (ICSD). The Open Crystallography Database (COD) provides an alternative free access to crystallographic information files.

### Photoelectrochemical experiments

We produced a pH 13.6 solution by preparing a 1 mol L ^-1^ sodium hydroxide electrolyte. The stock solution was prepared by adding 40 g of NaOH to 1 L Milli-Q ^®^ water (18.2 MΩ·cm). The electrolyte is pumped through the cell with an average flow rate of 1 mL min ^-1^ using a Harvard Apparatus PHD ULTRA Syringe Pump. We use 50 mL plastic syringes and cannulas, 0.8 mm in diameter that are squeezed into PTFE tubes of 0.7 mm in diameter. The tubes are connected to the *operando* cell using flangeless tube end fittings (Vici) that are screwed into M5 threads.

The electrochemical cell hosts three electrodes in which the FTO substrate serves as working electrode (WE), a Pt-wire acts as counter electrode (CE) and a Radiometer REF321 (Ag/AgCl/KCl 3 M) is the reference electrode (RE). The Pt-wire has 0.5 mm Ø, with a ~7.3 mm long electrolyte immersed segment providing 1.4 mm² or 0.014 cm² electrolyte exposed geometrical surface area. The reference potential of 212 mV vs standard hydrogen electrode (SHE) was converted onto the RHE (reversible hydrogen electrode) scale using the Nernst equation. The three electrodes are electrically connected using clamps. The current-voltage (I-V) experiments were realized using either a BioLogic VMP3 or a portable BioLogic SP-150 potentiostats. A potential range of -2.5 to +2.5 V is chosen, resulting in a 100 µV resolution. A fixed current range of 1 mA and a bandwidth factor of 5 is selected. The current average over 0.1 s is recorded. Solar-like irradiation was provided either by an Oriel solar simulator equipped with a 300 W Xe lamp which light emission was spectrally adjusted to resemble an AM 1.5 G spectrum using a dedicated filter or by a Lumixo-S plasma lamp (Lumartix) providing illumination closely resembling the AM 1.5G spectrum without need for spectral adjustment. Light intensity stemming from the Oriel solar simulator was further reduced with a neutral density filter (Newport, FS−ND). For both light sources, final intensity calibration was achieved by placing the PEC cell at a distance that provided a power density of 100 mW/cm ^2^ previously determined using a calibrated Si-photovoltaic reference cell (Oriel, 91150V). The illuminated surface area on the photoelectrode was 0.280 cm² and 0.031 cm² for in-house and *operando* cell, respectively. For *operando* experiments an optical fibre was used to direct the light from the solar simulator onto the photoelectrode. We thoroughly rinsed all components in contact with the sodium hydroxide solution (electrodes, cell interior, Si/Si _3_N _4_-chip) with deionized water after each use. Raw data and the explicit data treatment using OriginLab Pro are available in *Underlying data* ^30^.

### *Operando* cell

Technical drawings were created using the Autodesk Inventor 2017 software. FreeCAD is a freely available software alternative to view the 3D CAD files. The cell is machined from poly(methylmethacrylate) (PMMA) and additionally equipped with Si _3_N _4_ windows. To this end, the two Si _3_N _4_ membranes (1.2 mm (width) × 1.3 mm (length) × 500 nm (height)) are embedded into a 15 mm x 15 mm Si-chip (Norcada Inc). The technical drawings are provided as CAD files in *Underlying data* ^30^. The CAD files are provided as .ipt & .stp (identical to .step). The latter can be imported using FreeCAD.

### HERFD-XAS

Experiments were carried out at ID26 at the European Synchrotron Research Facility (ESRF) in Grenoble, France. To obtain Fe K-edge X-ray absorption spectra the incident beam was monochromatized using a double crystal monochromator with Si (111) crystals and tuned around the absorption edges of Fe and Ni, at 7112 eV and 8333 eV, respectively. The X-ray fluorescence was detected using a wavelength-dispersive Johann-type spectrometer in Rowland geometry which operated with five spherically bent Ge (440), Ge (620) or Si (620) crystals, to selectively reflect and focus Fe Kα _1_-, Kβ _1,3_- or Ni Kα _1_ radiation onto an avalanche photodiode (APD) detector. The total energy resolution given as the full width at half maximum (FWHM) of the elastic peak was 1.3 eV for Fe Kα _1_, 1.4 eV for Fe Kβ _1,3_ and 1.6 eV for Ni Kα _1_. A typical HERFD-XAS scan was measured from 7100 eV to 7200 eV for the Fe K-edge and from 8325 eV to 8414 eV for the Ni K-edge with a step size of 0.1 eV and a duration of 60 s per scan. All scans were corrected for the APD dead time and were normalized with respect to the beam intensity and total spectral area (TSA), being determined *via* the trapezoid rule, using PyMca 5.5.4 and OriginLab Pro, unless noted otherwise. Raw data and the explicit data treatment using PyMca and OriginLab Pro are available in *Underlying data* ^30^. To improve the signal-to-noise ratio (SNR) in *operando* experiments we averaged over 30 scans.

## Results and discussion

### *Operando* cell design and assembly

**Figure 2.**
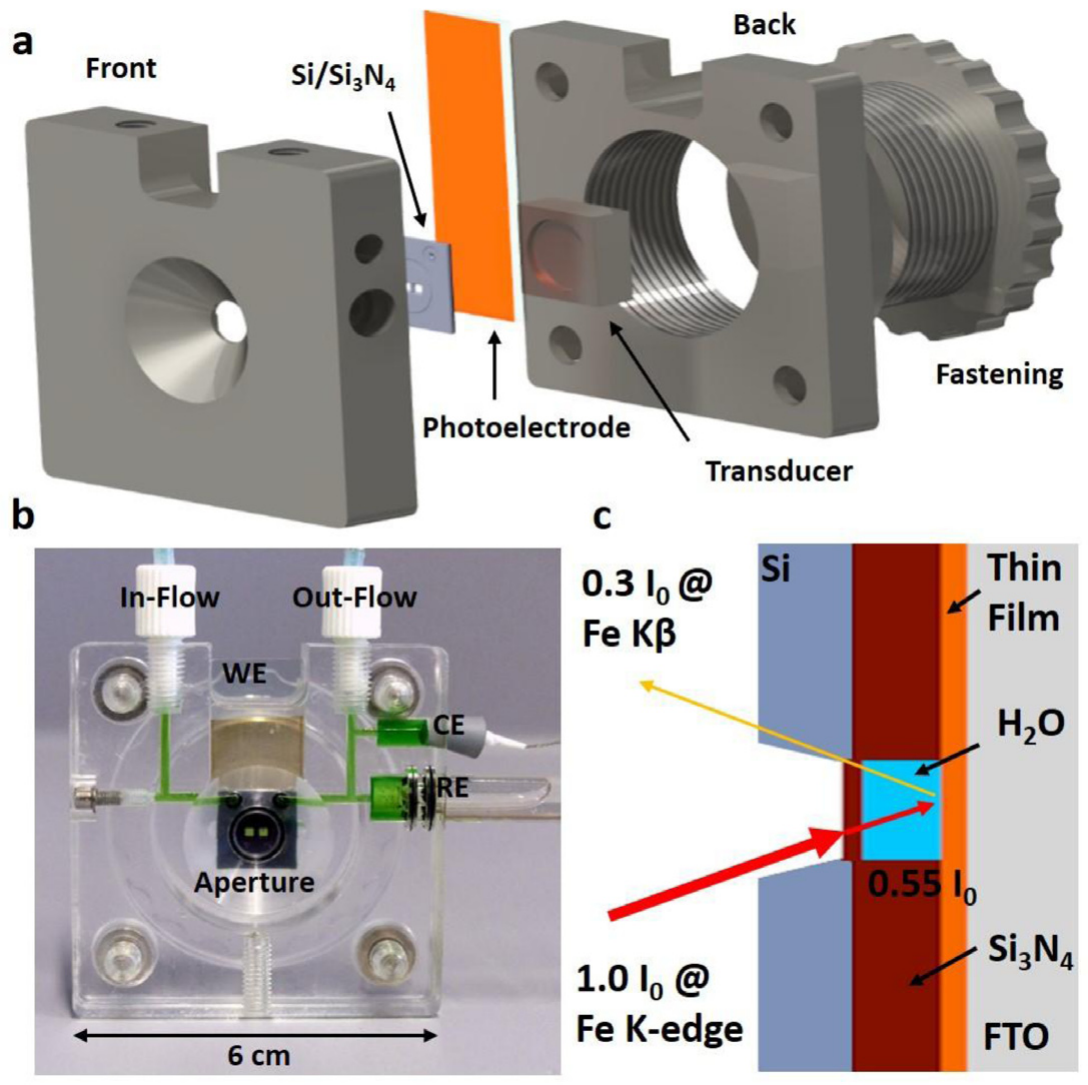
The schematics of the cell used for *operando* hard X-ray photon-in photon-out spectroscopy studies under photo-electro-chemical water splitting conditions. ( **a**) The exploded view highlights the most important components such as the front, transducer, back and fastening parts (dark grey), Si/Si _3_N _4_ chip as optical aperture for UV-Vis and X-ray beam (light grey) and photoelectrode (orange). ( **b**) Photograph of the cell showing the three electrodes configuration: thin-film based photoelectrode as WE, platinum wire as CE and Ag/AgCl electrode as RE. A green-colored solution is used to visualize the electrolyte path inside the cell. ( **c**) A schematic illustrating the optical path in a photon-in-photon-out process. The X-ray intensities incident on the sample and on the detector are given after passing 500 nm of Si _3_N _4_ and 400 µm of water for the Fe K-edge upon absorption (photon-in at 7112 eV) and for the Fe K *β*_1,3_ line upon emission (photon-out at 7059 eV).

The underlying idea was to conceive a measurement cell that provides consistent instrumental conditions, such as electrical connection and optical path length to explore, in a reproducible way, aqueous photo- and electrochemical processes occuring in thin film materials. The cell is dedicated for the use of conductive substrates such as fluorine-doped tin oxide (FTO) or indium tin oxide (ITO). It consists of six main parts which are front, back, fastening and transducer as well as Si/Si _3_N _4_-chip and photoelectrode as shown in Figure 2 a–b. The front part accomodates in- and out-flow connections, electrode slots, a mold to insert the photoelectrode and the Si/Si _3_N _4_ chip as well as the aperture. The front part is joined with the back part, which contains a threaded fastening. The transducer transfers the exerted pressure and reduces shear forces onto the photoelectrode and Si/Si _3_N _4_ chip. This architecture employs only mechanical forces for sealing and hence allows a rapid sample exchange. This decisive feature decouples sample preparation, i.e. the applied thin film deposition technique, from cell assembly and allows to probe thin films fabricated from physical and chemical methods. Additionally, the straightforward mechanical photoelectrode insertion reduces the necessarily needed and often costly Si/Si _3_N _4_chip to a single and reusable quantity. This is significantly more practical and inexpensive opposed to approaches where the sample is directly deposited onto the Si _3_N _4_ membranes ^23^. The sealing between the thin film and Si-chip is realized by compression and therefore it has to be ensured that films have even surfaces. The cell is designed to operate in a typical three electrode configuration, in which the photoelectrode acts as the working electrode (WE) and its operating voltage is measured against a reference electrode (RE). A commercially available Ag/AgCl electrode is chosen as RE which is encapsulated by a glass body that is inserted into the cell’s RE-slot and sealed with a pair of O rings as shown in Figure 2 b. The counter electrode (CE), a Pt-wire, compensates the current generated at the WE and is tightened by a rubber conus and a teflon tape. Principally, the RE and CE can be exchanged for measurements in electrolytes of different pH-values or for anodically more active electrodes, respectively. The cell’s front and back parts (dark grey) shown in Figure 2 a are manufactured by computer numerical control milling from poly(methylmethacrylat) (PMMA). PMMA provides the necessary water resistivity to perform aqueous photoelectrochemical experiments and transparency, which allows to spot possible electrolyte leakage and to follow the wetting behavior of the three electrodes. Especially the RE requires uniform electrolyte coverage of the porous frit, undisturbed by gas bubbles, to ensure a stable reference potential. Electrolyte is supplied from syringe pumps *via* chemically resistant poly(tetrafluorethylene) (PTFE) tubings. They are connected to the cell’s flow system *via* flangeless tube end fittings that ensure reliable, leakage-free operation. The cell’s channels have a diameter of 500 µm and are sufficiently wide for flow rates up to 5 mL min ^--1^. A flow cell is advantageous to a static one as a constant temperature is maintained during solar and X-ray beam irradiation reducing possible radiation damage. Additionally, continuously refreshing the electrolyte, which is consumed during water splitting, ensures a stable pH and moreover, releases the gaseous endproducts, H _2_ and O _2_. For mechanical stability and precise positioning, a M5 thread is implemented into the cell’s front part to mount the cell onto the beamline’s sample stage. The CAD files are provided in *Underlying data* ^30^.

Central in photon-in-photon-out spectroscopy is to minimize absorption losses for both the incident and fluorescent X-rays as schematically shown in Figure 2 c. Consequently, a very thin optical window material that consists of low atomic number (Z) elements is required which is also transparent in the visible region. Thus, we employed two 500 nm thin Si _3_N _4_ membranes as a windows, which are thick enough to provide the necessary mechanical stability to withstand the water pressure. They are embedded into a 400 µm thick Si chip which contains a fluidic channel that connects the fluid ports to the central 2-window area. The fluidic channels are defined by a 400 µm deep spacer, integral to the chip structure. The spacer layer is made of a multilayer of Si and SiN _x_ films. Although an even thinner electrolyte layer would minimize absorption effects, it might also introduce electrochemical mass transfer and flow rate limitations. Taking transmission losses into account ^36^, we estimated that this setup retains 30 % of the original intensity ( Figure 2 c) on detecting Fe K *β* fluoresence (7059 eV) while exciting at the Fe K-edge (7112 eV), as shown in detail in Section 1 of the SI and visualized in Figure S1 (see *Extended data* ^30^). The OriginLab project file used to plot the tabulated values is provided in *Underlying data* ^30^.

### Metal oxide photoelectrodes

We select hematite and transition metal ferrites photoelectrodes for the applicability tests of an *operando* HERFD-XAS cell. We choose hematite, because it is one of the most characterized metal oxide photoanode for water oxidation and served as model compound for *in situ* XAS and XPS studies ^23, 37^. Transition metal ferrites of the composition *M*Fe _2_O _4_ ( *M* = Mn, Co, Ni & Zn) are ternary oxides that offer to tune the optoelectronic properties by varying the non-iron cation ^38^. Similary to hematite, they have shown chemical stability for hours of operation ^39^ and are promising photoelectrochemical materials with an optical band-gap range between 1.5 eV and 1.9 eV, that enable theoretical, assuming 100% incident-photon-to-current (IPCE) conversion, current densities from 29 mA cm ^-2^ to 18 mA cm ^-2^ ^40^. Among the transition metal ferrites nanoparticles that are accessible *via* the benzyl alcohol route ^26, 27^ MnFe _2_O _4_ and CoFe _2_O _4_ demonstrated only marginal photocurrents and thus we exclusively focus on ZnFe _2_O _4_ and NiFe _2_O _4_ with Fe _2_O _3_ as the model photoelectrodes for *operando* HERFD-XAS studies, see Figure S2 ( *Extended data* ^30^). for corresponding photocurrent measurements. Additional information on crystal structure and thin film morphology including its thickness can be found in *Extended data* ^30^, section 2 Figure S3–S6.

### Photoelectrochemical measurements

Inferring mechanistic information from X-ray spectral changes relies on the controllable and predictable behavior of the applied stimulus i.e. electrical potential and solar illumination and their measurable effect. Thus, we compare the *operando* cell to a commonly used standard laboratory photoelectrochemical cell ^41^. Conducting photoelectrochemical experiments on the same material but in two different cells should reveal apparatus-related differences. Our analysis focuses on the working electrode potential regulation and photocurrent measurements.

**Figure 3.**
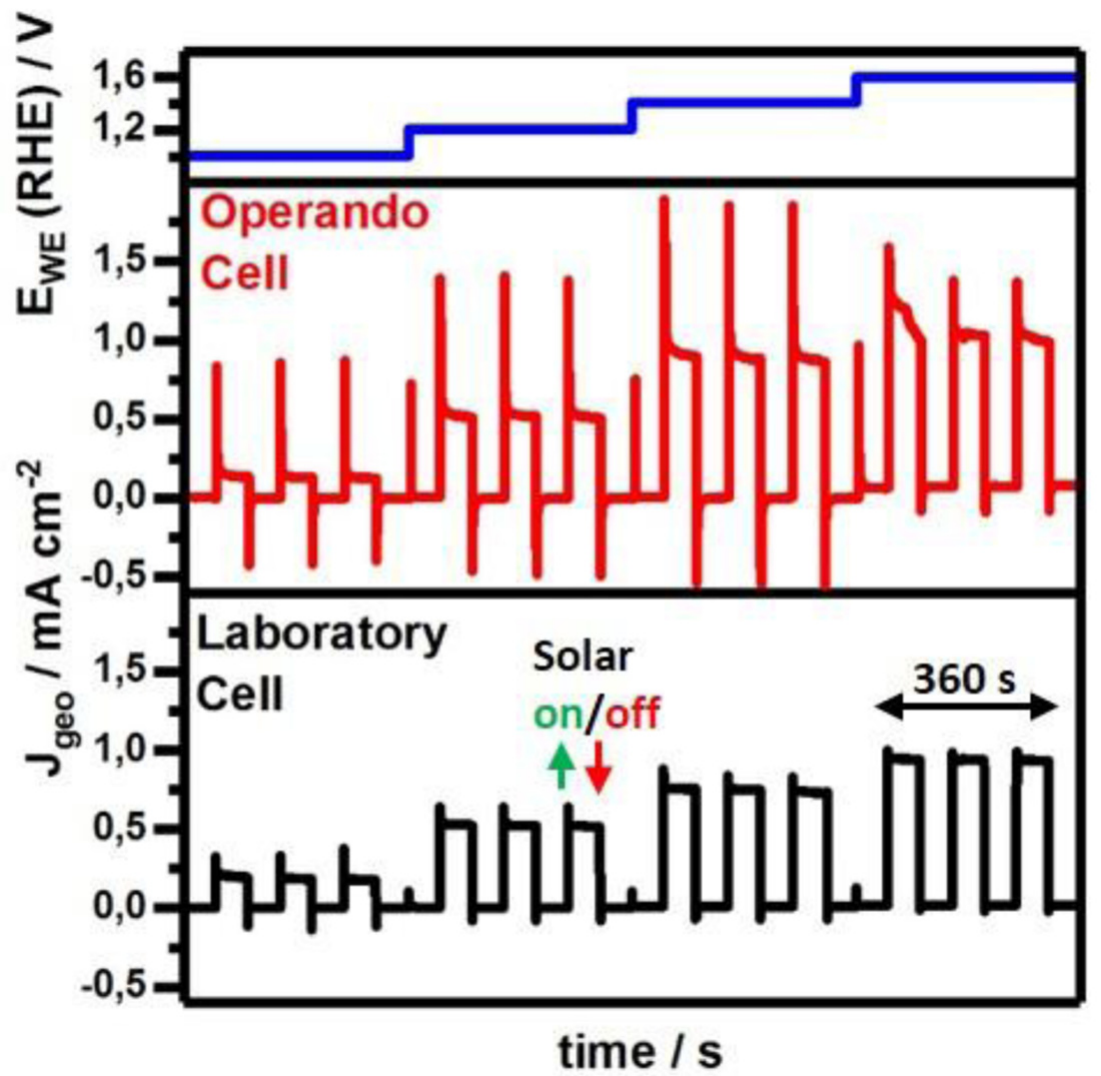
Comparison of photocurrent densities of the Fe _2_O _3_ photoanodes under constantly applied potentials (blue) recorded in the *operando* flow cell (red) and laboratory cell (black). Photocurrent measurements show that the *operando* cell generates low noise, and the photocurrent densities J _geo_, normalized to the geometrically illuminated area, are similar to those obtained in the laboratory cell.

Therefore, we compared constant potential or chronoamperometric measurements of Fe _2_O _3_ photoanodes in our *operando* cell with 0.031 cm ^2^ illuminated area versus our laboratory cell with 0.28 cm ^2^ illuminated area as shown in Figure 3. The measurement protocol includes a stepwise potential increase after 360 s during which dark and illuminated periods alternate every 60 s. The applied potential follows the set potential from 1.0 to 1.6 V vs RHE in steps of 0.2 V and demonstrates the successful voltage regulation ( Figure 3, top). In the dark, recorded current densities are 0.001–0.005°mA°cm ^-2^ for both cells in the applied potential range of 1.0 V to 1.4 V indicating no significant faradaic processes. At 1.6 V the applied potential starts to match the kinetic overpotential causing water oxidation with a rate up to 0.07 mA°cm ^-2^ in the *operando* cell, which is about 0.05°mA°cm ^-2^ (~3.5 times) greater than for the laboratory cell with 0.02 mA cm ^-2^. However, contrary to the laboratory cell which provides equally sized dark and illuminated areas on the photoelectrode, the *operando* cell’s total electrolyte exposed geometric surface area towards the photoelectrode amounts to 0.172 cm ^2^ (Figure S7 in *Extended data* ^30^) and is about 5.5 times larger than the illuminated area. Therefore, renormalization corresponding to a current density reduction by a factor of 5.5 to 0.013 mA cm ^-2^ reveals current densities in the laboratory cell to be 1.6 times higher. During illumination and under potentials of 1.0 V vs RHE values stabilize at 0.25 mA cm ^-2^ and are doubled to 0.5 mA cm ^-2^ upon potential increase to 1.2 V. Within this voltage window the photocurrent densities recorded in our *operando* cell match very well the values obtained in the laboratory cell. However, by increasing the applied potential further to 1.4 V and 1.6 V the *operando* cell delivers slightly higher steady-state photocurrent densities of about 0.90 mA cm ^-2^ and 1.05 mA cm ^-2^, respectively than the laboratory cell with 0.75 mA cm ^-2^ and 0.95 mA cm ^-2^, respectively. Whether in the dark or under illumination, recorded current densities deviate at higher applied voltages for which the effect of potentially different solution resistances would be amplified. Further contributing factors are related to variations during sample preparation ^42^ or potential sample modification under operation ^43, 44^, both itself promising research topics but difficult aspects to control due to the material’s physical complexity. However, electrolyte mass transfer behavior will clearly be affected as the cell's geometry is modified and liquid flow is actively driven compared to the static laboratory cell. Up to now, we considered steady-state values varying maximally about 1.6 times but observe 7–8 times variation in transient peak current densities upon triggering solar illumination or a potential step (Figure S8 in *Extended data* ^30^). Such transients are common in chronoamperometric experiments in the dark ^45^ and under illumination ^46, 47^ and are explained *via* non faradaic charging processes, both within the semiconductor and electrolyte, or *via* electrolyte diffusion after initial near-electrode depletion. As the same transient behavior is observed for measurements on a different material, namely NiFe _2_O _4_ (Figure S9 in *Extended data* ^30^), the underlying cause might be cell geometry and flow.

The previous discussion is a starting point to understand the *operando* cell’s photoelectrochemical characteristics. In future, more quantitative work will rely on comprehending its current-voltage behavior in detail particularly regarding uncompensated solution resistance lowering the electrical potential the WE actually experiences. However, the near steady state conditions are reached within seconds after the external stimulus (light or bias) and can be reproduced multiple times for extended periods ( Figure 6) allowing repeatable XAS scans to be averaged, as discussed in the next section. Therefore, we conclude that photoelectrochemical conditions are maintained that closely resemble laboratory testing conditions and allow to qualitatively investigate associated phenomena of thin film photoelectrodes using X-ray absorption spectroscopy.

In addition, the operation in continuous flow provides significant advantages. We maintain a constant temperature and thus can exclude thermally induced effects due to UV-Vis and X-ray absorption as opposed to photo-electro-chemical effects. In the same instance, we avoid the formation of gaseous bubbles that can adsorb onto the photo-electrodes surface and decrease the active surface area, which would lower the photocurrent. Additionally, as gas droplets are of lower density than water, they enhance transmission of both UV-Vis light and X-rays. Frequent density variations along the X-ray beam path lead to abrupt, spike like intensity fluctuations and generate unreliable data that cannot be normalized for. Consequently, by minimizing gas accumulation our *operando* cell raises the amount of high-quality valid data.

### Data quality under *operando* conditions

Assuming the energy resolution is unaffected by measuring under *operando* conditions, the signal-to-noise ratio (SNR) is an important parameter characterizing the data quality. Here, the SNR is described as quotient of the arithmetic mean *μ* and the standard deviation σ ^48, 49^ and is proportional to the square root of the signal obtained under identical conditions ^50^ either *via* prolonged measurement or *via* averaging of consecutive measurements ^51^. We assume non-correlated measurements with time invariant white noise to be present in the measured spectral range ^52^. To calculate the SNR the spectral region from 7180 eV to 7184 eV was chosen, in which the measured intensity was almost constant. To assess the additional time required to compensate for absorption losses caused by the electrolyte and the Si _3_N _4_ windows, we estimated a relative signal detection yield of 30% upon Fe K-edge absorption and Kβ-fluorescence ( Figure 2 c, *Extended data* ^30^ Section 1 and Figure S1), which corresponds to a SNR decrease by a factor of 1.8.

**Figure 4.**
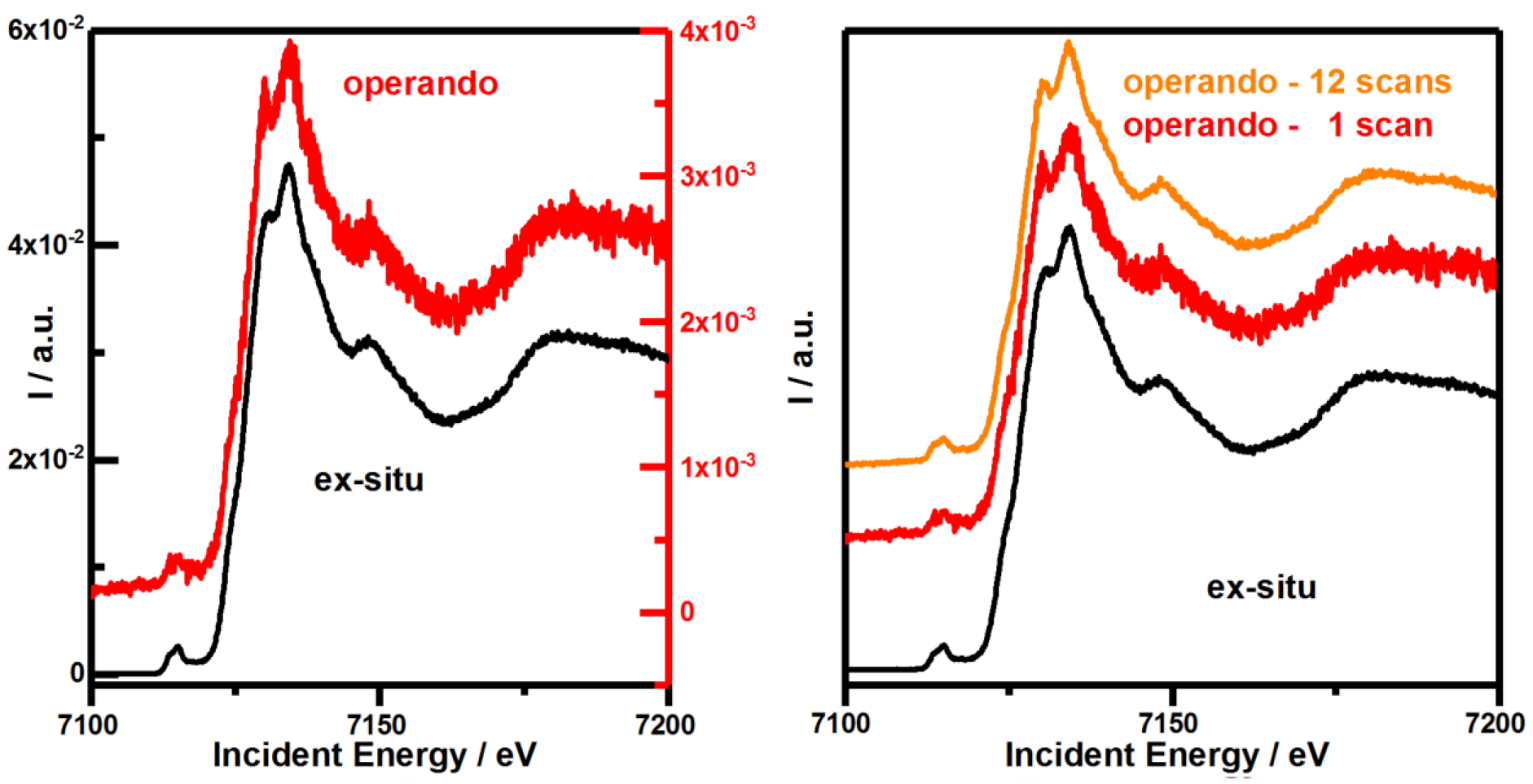
Comparing the quality of Fe K-edge spectra on Fe _2_O _3_ obtained *via* Kβ-fluorescence detection under *ex situ* and *operando* conditions. The sample was measured under *ex-situ* (black) and *operando* (red & orange) conditions. Left: Single scans are recorded for 60s and normalized to incident beam intensity to compare signal intensity. Right: Scans are normalized to incident beam intensity and total spectral area (TSA) to compare the signal-to-noise ratio (SNR). Please note the different black and red scale bars corresponding to the same colored data traces in the left figure.

We compare two Fe K-edge scans on an approximately 50–80 nm thin Fe _2_O _3_ film under *ex-situ* ( Figure 4, left black) and *operando* conditions ( Figure 4, left red). The intensity of the Fe K-edge spectrum measured in *operando* is attenuated by a factor of 11.68, representative of a detection yield of 8.56%.

**Figure 5.**
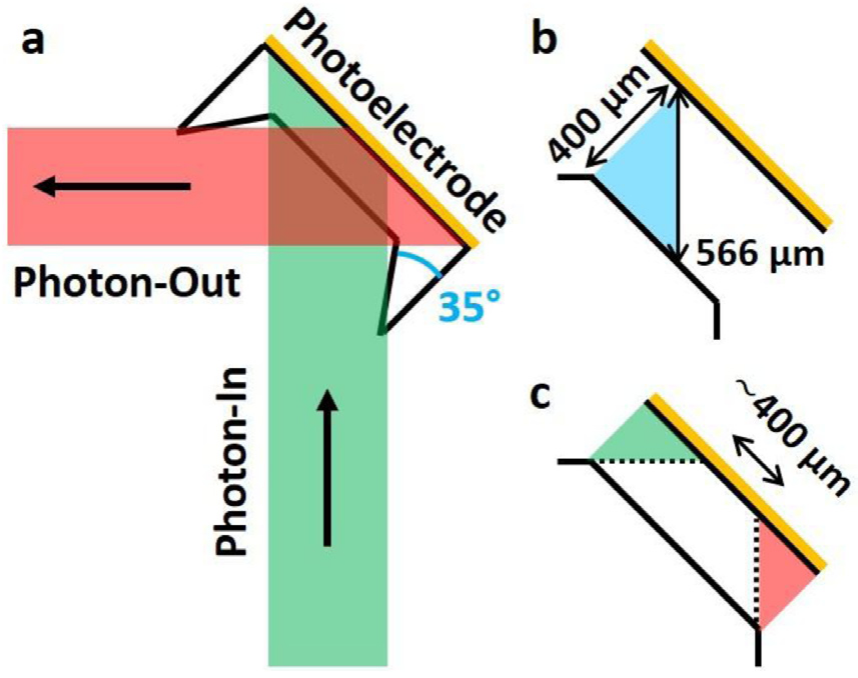
( **a**) Schematic (top view) illustrating photon-in (incident X-ray, green) and photon-out (fluorescent X ray, red) beam profiles covering different regions on the photoelectrode (orange). Intensity is collected from a photoelectrode (orange) region where photon-in and photon-out profiles overlap. Profiles are defined through incident beam size and crystal spectrometer focus size. ( **B**) Orthogonality between photon-in and photon-out beam prolongs the optical path length in the electrolyte to 566 µm. ( **C**) The overlapping region of photon-in and photon-out profiles is reduced to approximately 400 µm.

To explain the deviation between estimation and experiment we need to consider the beam’s spatial profile and the measurement geometry ( Figure 5 a). The intensity loss is a result of the orthogonality between the photon-in and photon-out beam. First, it increases the optical path length inside the electrolyte ( Figure 5 b) and second it leads to a smaller mutual focal spot of photon-in and photon-out beam ( Figure 5 c). The optical path length is enlarged from 400 µm to 566 µm which explains a lower relative detection yield of 18.7% (Extended data) but not as low as 8.56%. This difference can be explained considering that the photon-in beam size of 0.8 mm (w) × 0.1 mm (h) yields a greater projected beam size of 1.13 mm (w) × 0.1 mm (h) on the photoelectrode which reduces the average photon flux by 0.707. Although in principle the spectrometer focus of all 5 crystals (~ 1–2 mm, determined from alignment scans) is large enough to cover the area the orthogonal detection geometry ( Figure 5 a) allows to cover only a width of ~ 0.4 mm ( Figure 5 c). This represents a relative detection yield of 35.4%. In combination with previously determined losses due to the extended optical path length an overall relative detection yield of 6.6% is calculated which approximates the experimentally determined 8.56% detection yield. Thus, the SNR of a single *operando* scan compared to an *ex-situ* scan should be reduced by 3.42, square root of 11.68, but is determined to be only lowered by 3.06 ( Figure 4, right and OriginLab project file in *Underlying data* ^30^). Hence, our assumptions about non-correlated measurements and underlying white noise might not be entirely correct. An average out of 12 *operando* scans therefore slightly exceeds the SNR of a level of an individual *ex-situ* scan (OriginLab project file in *Underlying data* ^30^). Thus prolonged measuring or averaging consecutive measurements is effective in restoring the initially SNR of *ex-situ* measurements. Our theoretical estimation seems to capture most essential aspects that cause this decline and thus provides understanding as well as guidelines to optimize the SNR for future *operando* studies. Thus, our cell can be directly used at extremely brilliant source (EBS) storage ring like MAX IV, ESRF or after the upgrade of Petra IV at the time scales relevant for photoelectrochemical processes.

Prior to operando experiments, *ex-situ* HERFD-XAS measurements shown in Figure S10 (see *Extended data* ^30^) were performed and provide no evidence for X-ray beam induced spectral changes often called “beam damage”. Finally, we conduct the *operando* experimental protocol and record HERFD-XAS spectra of hematite, NiFe _2_O _4_ and ZnFe _2_O _4_ (Figure S11 in *Extended data* ^30^) under open-circuit (OC), electrochemical (EC) and photoelectrochemical (PEC) conditions. The Fe K edge excitation spectra on hematite (Figure S11 a in *Extended data* ^30^) are recorded *via* the Kβ fluorescence line (7059 eV) and spectra on NiFe _2_O _4_ and ZnFe _2_O _4_ (Figure S11 b-c in *Extended data* ^30^) are measured *via* the Kα fluorescence line (6405 eV) to increase the signal intensity. Note that we also recorded Ni K-edge spectra on NiFe _2_O _4_ (Figure S11 d in *Extended data* ^30^). Interestingly, despite high quality of the data, at minutes time scale we do not observe any changes under PEC conditions, neither in the pure HERFD-XANES spectra nor in the differential spectra (ΔEC and ΔPEC) as shown in Figure S11 (see *Extended data* ^30^). However, we want to stress that this is not a necessary premise to conclude that we nevertheless demonstrate the general applicability of our cell to measure high quality (high SNR) HERFD-XAS spectra under photoelectrochemical conditions. We provide sufficient evidence to support claims on all of the cell’s capabilities. Therefore, a scientific apparatus is presented that allows to correlate independent variables (voltage, solar illumination) with dependent variables such as the material’s current and X-ray emission response. This study only tested an infinitesimally small part of the available parameter space to prove the cell function’s principal validity. The experimental conditions can easily be extended towards more intense or pulsed light sources and pump-probe detection schemes to study PEC effects at shorter time scales for example using time-resolved X-ray absorption spectroscopy ^53^.

### Electrochemical X-ray absorption detection

**Figure 6.**
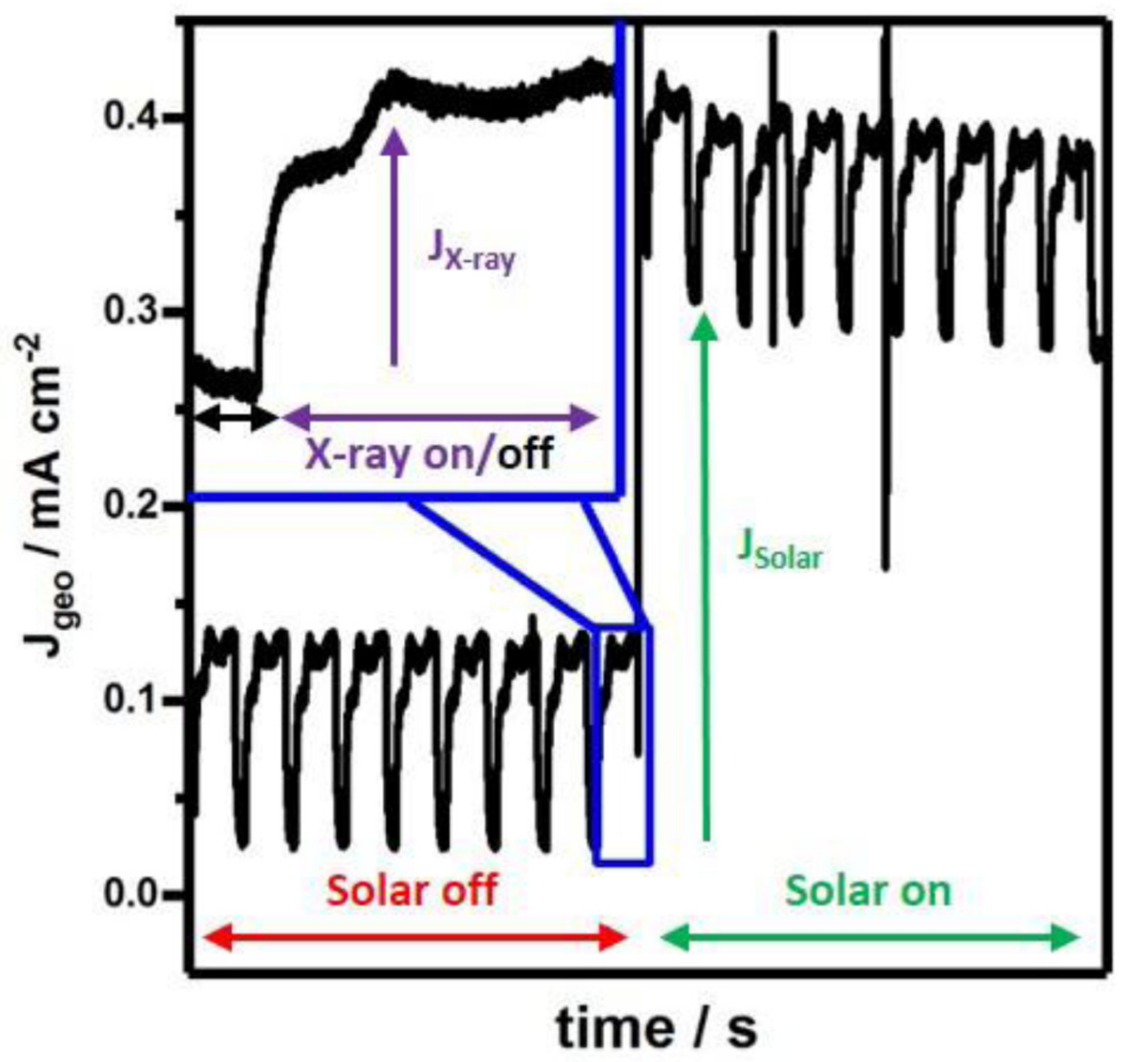
Constant potential photocurrent measurements, based on the geometrically illuminated area of Fe _2_O _3_ photoanodes at 1.6 V vs RHE recorded in the *operando* flow cell. (Inset) During HERFD-XAS at the Fe K-edge X-ray exposure induces additional current suitable for ionic-current XAS detection or to examine charge carrier transport properties.

The X-ray fluorescence (XRF) that is detected during the experiment is one of many secondary processes that follow the initial X-ray excitation. Additionally, to a radiative decay there are also non-radiative secondary processes that generate electronic or ionic currents, which can be used to measure X-ray absorption spectra. Under applied bias conditions we can distinguish the photoelectrochemical current from the periodically repeated X-ray beam induced current ( Figure 6) caused by individual Fe K-edge scans. By zooming into one of the respective sections the time-dependent current profile resembles the X ray absorption profile monitored *via* fluorescence detection ( Figure 6, inset). Since the measured current flows mainly between the thin film photoanode working electrode and platinum wire counter electrode, ionic charge opposed to electronic charge will be carried across the electrolyte in between both electrodes. This has lately been demonstrated as a more bulk-sensitive detection mechanism than total electron yield (TEY) or total fluorescence yield (TFY) and has been named total ion yield (TIY) ^54, 55^. It might be of use when probing the full depth of µm thick films where redshifted and escaping fluorescent photons are strongly attenuated but electrical current can still be recorded.

Instead of simply considering the current as a secondary signal that serves to record X-ray absorption spectra, it can also be applied to investigate electronic transport properties using methods that are referred to as photoconduction ^56^ or X-ray beam induced current (XBIC) ^57^. The latter technique was used to spatially map bottlenecks of low minority carrier diffusion length in multicrystalline silicon solar cells caused by metal impurities such as iron ^58^. A short-wavelength X-ray beam can provide a much smaller illumination cross-section compared to a beam of visible-wavelength light that offers high spatial resolution and element selectivity. Both characteristics have been taken advantage of to visualize and correlate the XBIC with the elemental composition of GaAs across a single nanowire ^59^. These examples illustrate prospective experiments with the *operando* cell in which electrochemical current detection upon element selective X-ray excitation would allow to determine atom dependent X-ray beam induced currents such as in multinary oxides, e.g. ZnFe _2_O _4_, or doped semiconductors as for example Ti-doped Fe _2_O _3_. Thus, deeper insights into individual atomic contributions to electronic transport properties would be provided.

## Conclusions and outlook

The *operando* X-ray absorption spectroscopy approach generally offers insight into device operation but requires a sophisticated sample environment. Studying thin films in photoelectrochemical water splitting cells poses obstacles for XAS under working conditions, as many interdependent parameters and functionalities have to be optimized. Our work approaches this challenge based on thorough estimations of radiative transmission leading to an *operando* HERFD-XAS flow cell in which the electrolyte thickness is reduced to a minimum. Consequently, the cell delivers high transparency for both visible-light (1.5–3 eV) and X-ray (≥ 6400 eV) radiation. The resulting high signal-to-noise ratio enhances the detection limit and minimizes the synchrotron data collection time. Moreover, by reusing a single Si _3_N _4_/Si chip for multiple measurements the experimental cost is greatly reduced. PEC current and voltage can routinely be determined and controlled using a standard 3 electrode configuration connected to a commercially available potentiostat that allows to conduct numerous aqueous electrochemical experiments with low electrochemical noise. The high electrochemical and X-ray spectroscopic signal quality are prerequisite to take advantage of the cell’s central aspect which is the facile thin film exchange mechanism that relies purely on mechanical fixation. Our approach treats the X-ray transparent window and thin film separately. Thus, the sample can be independently prepared and optimized. This feature made it possible to collect a comprehensive dataset on three different metal-oxide photoelectrodes during a single synchrotron beamtime (see *Underlying data* ^30^) and can in principal be extended to any desired thin film material. Finally, we describe the potential to electrochemically measure X-ray absorption spectra that might be a suitable detection method for lower X-ray energies. Alternatively, recording the X-ray beam induced current (XBIC) that is element-specific might layout atomic contributions to photocurrents, possibly with high spatial resolution using an X-ray microprobe. Overall, this work targets the instrumental bottleneck in *operando* fluorescence-detected X-ray absorption spectroscopy by establishing a measurement routine for experiments on photo- and electrochemical thin film devices, that significantly simplifies the technique’s applicability and will stimulate further research.

## Data availability

### Underlying data

Zenodo: Dataset for the development of a "Flow cell for operando X-ray photon-in-photon-out studies on photo-electrochemical thin film devices". https://doi.org/10.5281/zenodo.6560384 ^30^.

This project contains the following underlying data:

- CAD_IPT.zip (the cell’s 3D technical drawing files in the specific AutoDesk Inventor format as .ipt-files for individual parts and .iam-files for the assembly).
- CAD_STP.zip (the cell’s 3D technical drawing files in the .stp or .step format “STandard for the Exchange of Product model data”. As an ISO standard it is very accessible).
- GIXRD.zip (the GIXRD raw data on the thin film materials as .xrdml, .csv and .xy files and the OriginPro project file in which data was processed and plotted.).
- HERFD.zip (the HERFD-XAS raw data on the thin film materials conducted during ex-situ and operando measurements as unspecified files to be opened *via* PyMca and the OriginPro project file in which data was processed and plotted.).
- PEC-duringHERFD.zip (the photoelectrochemical raw data on the thin film materials conducted during operando measurements as .mpr and .mpt files, the former to be opened *via* the BioLogic software and the latter by any scientific data processing software. It also contains the OriginPro project file in which data was processed and plotted.).
- PEC.zip (the photoelectrochemical raw data on the thin film materials conducted during ex-situ measurements as .mpr and .mpt files, the former to be opened *via* the BioLogic software and the latter by any scientific data processing software. It also contains the OriginPro project file in which data was processed and plotted.).
- SEM.zip (the SEM image on the thin film materials after brightness and contrast enhancement and cropping).

### Extended data

Zenodo: Dataset for the development of a "Flow cell for operando X-ray photon-in-photon-out studies on photo-electrochemical thin film devices". https://doi.org/10.5281/zenodo.6560384 ^30^.

This project contains the following extended data:

- ExtendedData.zip (additional information arranged in a word document that are directly linked to the main manuscript such as figures).

Data are available under the terms of the Creative Commons Attribution 4.0 International license (CC-BY 4.0).

## Ethics and consent

Ethical approval and consent were not required.
