# Supplementary material for: Flow cell for operando X-ray photon-in-photon-out studies on photo-electrochemical thin film devices
Source: Open Res Eur. 2022 Dec 23;2:74. Originally published 2022 Jun 7. [Version 2] doi: 10.12688/openreseurope.14433.2 (PMC10446061; doi:10.12688/openreseurope.14433.2)
Supplement: Peer review history

# Peer review: Reviewer response for version 2

**DOI:** 10.21956/openreseurope.16671.r30501

**Published:** 2023-01-13

**Authors:** Walter S. Drisdell

**Affiliations:** 1 Chemical Sciences Division, Lawrence Berkeley National Laboratory, Berkeley, CA, USA

I thank the authors for their detailed reply to my comments, and for the edits to the section discussing data quality for operando spectra collected in the cell. I can now recommend indexing. I also respond to some of the questions from the authors below:

 I brought up a parallel electrode configuration because this can be important for systems driven by an externally applied voltage (e.g. applying a voltage with the potentiostat). In that case, if the electrodes are not parallel, there will be a nonuniform electric field at the working electrode which makes it difficult to determine if the X-rays are actually probing the active part of the electrode. In a light-driven system like the one here, I suspect this concern is eliminated, because the electric field at the working electrode is controlled by the illumination. A non-parallel configuration will create a non-uniform electric field at the counter electrode, but that doesn't matter. So for studies of photoelectrochemical systems driven by light, as demonstrated here, this may not be a major concern.

 As for mass transport, this can be important for electrochemical reactions in which some of the reactants are solvated species. For example, in electrochemical CO2 reduction, dissolved CO2 is a reactant, and is present at low concentration due to its small Henry coefficient. In that case, it's easy to deplete the dissolved CO2 under operation, unless there is sufficient mass transport (usually via liquid flow) to replenish CO2 at the electrode surface. The cell design shown here would be sub-optimal for such a reaction due to (I presume) low flow rates and low mass transport. Also, many electrochemical reactions (including CO2 reduction) form gas-phase products as bubbles on the electrode surface, which can interfere with measurements if they cannot be swept away through liquid flow. The cell reported in this paper may not be ideal for such reactions, given (I presume) a low liquid flow rate due to the small flow channels. But for other reactions, this will not be a concern. So it depends on the application.

 Lastly, I thank the authors for elaborating on the reasoning for two small silicon nitride windows instead of one larger one. The in situ cells I have used featured single, larger silicon nitride windows, but we were not particularly concerned about water pressure. I wonder if the pressure in this cell might be higher due to the microfluidic liquid flow? Regardless, I appreciate the authors elaborating on the engineering decisions for this.

Is the rationale for developing the new method (or application) clearly explained?

Yes

Is the description of the method technically sound?

Yes

Are the conclusions about the method and its performance adequately supported by the findings presented in the article?

Yes

If any results are presented, are all the source data underlying the results available to ensure full reproducibility?

Yes

Are sufficient details provided to allow replication of the method development and its use by others?

Yes

Reviewer Expertise:

In situ synchrotron X-ray characterization, chemistry, electrocatalysis, energy materials.

I confirm that I have read this submission and believe that I have an appropriate level of expertise to confirm that it is of an acceptable scientific standard.

# Peer review: Reviewer response for version 2

**DOI:** 10.21956/openreseurope.16671.r30500

**Published:** 2023-01-11

**Authors:** Alessandro Minguzzi

**Affiliations:** 1 Dipartimento di Chimica, Università degli Studi di Milano, Milan, Italy

Dear Philipp Jäker,

 I acknowledge and appreciate your reply to my review of your interesting paper. 

 I think that your responses are fully satisfactory, and I have no other comments. In my opinion the paper can be considered at the end of its editorial path.

 For what concerns current cell versions, your conclusions based on our papers are substantially correct. In the latest version of our cell, the electrode is as close as possible to the mylar film to minimize the absorption from the solution. At the same time, a too thin layer of solution leads to not-negligible ohmic drops. It is unfortunately impossible for us to precisely determine the thickness of the solution (estimated in the range 100-300 microns), but this setup allowed us to successfully work at the Ni-K edge (Malara *et al.*, 2020 ^1^ and Tsyganok *et al.*, 2020 ^2^).

 If the studied reaction involves gas formation, the insulation of part of the sample area due to bubble retention is another factor to consider when a quite thin solution layer is desired. In this sense, your cell has undoubtedly the main advantage of guaranteeing an efficient bubble removal by a correct design of the solution flux.

Is the rationale for developing the new method (or application) clearly explained?

Yes

Is the description of the method technically sound?

Yes

Are the conclusions about the method and its performance adequately supported by the findings presented in the article?

Yes

If any results are presented, are all the source data underlying the results available to ensure full reproducibility?

Yes

Are sufficient details provided to allow replication of the method development and its use by others?

Yes

Reviewer Expertise:

electrochemistry, photoelectrochemistry, X-ray spectroscopies

I confirm that I have read this submission and believe that I have an appropriate level of expertise to confirm that it is of an acceptable scientific standard.

## References

[1] : Direct Observation of Photoinduced Higher Oxidation States at a Semiconductor/Electrocatalyst Junction. *ACS Catalysis* .2020;10(18) : 10.1021/acscatal.0c02789 10476-10487 10.1021/acscatal.0c02789

[2] : Operando X-ray Absorption Spectroscopy (XAS) Observation of Photoinduced Oxidation in FeNi (Oxy)hydroxide Overlayers on Hematite (α-Fe2O3) Photoanodes for Solar Water Splitting. *Langmuir* .2020;36(39) : 10.1021/acs.langmuir.0c02065 11564-11572 10.1021/acs.langmuir.0c02065 32900201PMC7586389

# Peer review: Reviewer response for version 1

**DOI:** 10.21956/openreseurope.15578.r30353

**Published:** 2022-11-14

**Authors:** Walter S. Drisdell

**Affiliations:** 1 Chemical Sciences Division, Lawrence Berkeley National Laboratory, Berkeley, CA, USA

The authors demonstrate a cell design for in situ X-ray absorption spectroscopy (XAS), particularly in high energy resolution fluorescence detection (HERFD) mode, on aqueous photoelectrochemical systems. The design emphasizes the ease of changing samples, so robust comparisons can be made between different catalysts within a single block of beamtime at a synchrotron facility. The design also specifically emphasizes solar light as a driver for the chemistry. Most in situ cells for XAS operate in the dark using externally applied voltages as the driver. Recent interest in direct photo-driven systems means a cell that accommodates solar light is desirable.

 The cell design and operation are demonstrated thoroughly, but there are clear downsides that come with the "quick swap" sample scheme. Generally speaking, electrochemical cells for in situ XAS must either bring the X-rays in through the back of the sample, or through the electrolyte before impinging on the front of the sample. This design does the latter, because this means samples are not directly deposited onto the expensive (and fragile) silicon nitride windows, and can therefore be swapped more easily. But even the thin (400 - 500 micron) liquid layer between the window and the sample introduces significant loss of signal, a roughly 70% loss compared to the ex situ measurement. This loss will get worse for any application looking at lighter metals as well. The authors demonstrate Fe K-edge data, but Cr K-edge or Ti K-edge would have worse signal loss.

 In addition, the thin liquid channels place constraints on the electrochemistry; for example, the counter electrode is not parallel to the working electrode sample, instead located within one of the sub-mm flow channels past the main cell volume. The cell also lacks a membrane, so Pt from the counter electrode could migrate to the sample surface (although the fact that the counter electrode is so far away may help with this). I suspect that there may be upper limits on mass transport as well, although these did not come into play in the demonstrations shown here, and are likely less important for light-driven systems than those run via external voltage. If the sample were deposited on the window instead, and the X-rays and solar light were brought in through the back, there would be no constraints on the liquid side of the cell, enabling a parallel electrode configuration and larger flow channels that can support high current densities. Also, there would be minimal attenuation of the X-ray signal by the electrolyte, since the electrolyte is on the far side of the sample.

 As I see it, the advantages to this cell are the ability to easily change samples, and that samples need not be partially transparent to X-rays or solar light (in other words, samples need not be thin films, although it's unclear if thicker samples can be mounted or if the size limitations effectively mean that thin films must be used). For some applications, for example screening large numbers of potential photocatalyst materials, this is a big advantage and this cell design will be useful. For other studies, using a "sample on window" design may be better because it gives better XAS signal and can support higher current density operation, even though sample preparation and swapping is more difficult.

 I do have one question about the design: I do not understand why two silicon nitride windows are used. Is there a separate window for input X-rays vs. detected fluorescence? Figure 5 suggests not. Is one window for the solar light while the other is for X-rays? It seems to me that a single window with larger area would work better, as is shown schematically in Figure 1. This is especially true given that Figure 5 shows that not all of the fluorescence can be extracted because the window is not large enough to cover the full solid angle. The discussion of this figure is confusing, in fact, because it mentions overlap between photon-in and photon-out beams, but in reality fluorescence is generated only where the input photons impinge upon the sample and can be detected as long as they can pass through the window at 90 degrees relative to the input beam. There is no "output beam" that matches the profile shown in Figure 5, that seems to more accurately describe which parts of the sample can be "seen" by the detector. Ideally, the detector should be able to "see" the entire illuminated area on the sample, but from this Figure it seems that is not the case in this design.

 A final minor correction: in the plain text summary, the phrase "within a signal device" should read "within a single device".

Is the rationale for developing the new method (or application) clearly explained?

Yes

Is the description of the method technically sound?

Yes

Are the conclusions about the method and its performance adequately supported by the findings presented in the article?

Yes

If any results are presented, are all the source data underlying the results available to ensure full reproducibility?

Yes

Are sufficient details provided to allow replication of the method development and its use by others?

Yes

Reviewer Expertise:

In situ synchrotron X-ray characterization, chemistry, electrocatalysis, energy materials.

I confirm that I have read this submission and believe that I have an appropriate level of expertise to confirm that it is of an acceptable scientific standard, however I have significant reservations, as outlined above.

# Response

**Published:** 2022-11-30

**Authors:** Philipp Jäker

**Affiliations:** ETH Zürich, Switzerland

Dear Walter S. Drisdell,

 We highly appreciate your encouraging and constructive report and generally agree with your comprehensive summary about the advantages and disadvantages of design considerations concerning the position of the thin film sample, e.g. front vs back with respect to incident X‑ray beam direction. I don’t quite understand the potential issue with the non-parallel placement of working and counter electrode. Mass transport limitations near the very small Pt wire could indeed be an issue but I would argue that the Pt surface area can still substantially and relatively easily be enhanced. At low to moderate mass transport limitations, the potentiostat is still able to compensate by increasing the counter electrode’s bias. Potentially more significant than mass transport is the uncompensated resistance of the working-reference-electrode circuit branch. On a rough back on the envelope calculation modelling the liquid channels as resistance elements might add about 100 mV to the working electrode to what currently is measured with respect to the reference electrode. It is very briefly mentioned following figure 3 that uncompensated resistance should be analyzed in future work. I agree that a membrane could provide protection against cross-electrode contamination both from the electrode material itself as well as from the water splitting reaction products. Hopefully, others will improve this cell design and further quantify some of its measurement properties. Regarding potential sample thickness and type: If one exactly reproduces this cell one will be constrained by the working electrodes (WE) (here FTO), width and length but not thickness to some extent. The working electrode slot within the cell currently is thicker than the FTO and so leaves room in the µm to mm range for thicker films. The liquid channel thickness is completely predefined within the Si/Si _3_N _4_ chip. The cell crucially relies upon a very smooth working electrode surface sealing the channel within the Si/Si _3_N _4_. If this is achieved one can also use different electrode materials as long as the principle width and length dimensions are considered. One can imagine using a smooth metallic electrode as back-side transparency is not relevant. Or use a metallic electrode to study photoelectrochemical processes within an X‑ray absorbing electrolyte. Regarding one vs two Si _3_N _4_:

 I agree that one larger Si _3_N _4_ window would render the experiment potentially simpler and increase signal detection. However, Norcada, the company responsible for design and manufacturing, recommended this window size as any further increase only increases the mechanic instability against the water pressure. I cannot provide you with anything more concrete or even quantitative. As Norcada improves their production process they might be able to produce larger windows with the same thickness but greater mechanical stability. Regarding the discussion of Figure 5: I agree that the discussion was a bit confusing and I thank you for your input especially regarding what we described as “photon-out” beam. Indeed, a more appropriate description than that is actually the Bragg crystal analyzer focus on the sample. I have now rewritten large parts of that section and hope it to be more understandable. But the previous conclusion remains in that there is an underlying mismatch between the spectrometer focus and the X‑ray excited area. Small X‑ray beam sizes, thinner electrolyte thicknesses or larger Si _3_N _4_ windows may even fully reduce it but all come with different disadvantages/consequences.

# Peer review: Reviewer response for version 1

**DOI:** 10.21956/openreseurope.15578.r29519

**Published:** 2022-06-29

**Authors:** Alessandro Minguzzi

**Affiliations:** 1 Dipartimento di Chimica, Università degli Studi di Milano, Milan, Italy

This is a valuable contribution that will be of high interest for synchrotron users and experts in photoelectrochemistry (in-situ/operando) XAS. I think it can be indexed as it is provided that the following comments are considered by the authors.

1. The sentence in the Introduction, “Current cell versions are limited by electrochemical noise or uncessarily high X-ray beam attenuation.” can be improved, since previous works (for example Fracchia *et al.*, 2019 ^1^) clearly show the effectiveness of other XAS cells for in-situ/operando experiments in photoelectrochemistry with no significant noise or X-ray attenuation.
2. For what concerns the total ion yield, I suggest a deeper discussion. Indeed, this ion current flow is the result of electrons ejected after X-rays hit the sample. Being the photoelectrode under a bias, the loss of these electrons is (or might be) compensated by electrons circulating in the external circuit (indeed, Fig. 6 reports an electric current) that are accompanied by ionic flowing in the electrolyte solution.
  This (photo)current is therefore both electronic and ionic, this is why I find that the term TIY is possibly not fully appropriate. However, I do agree that monitoring this signal might represent a very interesting new source of information! Moreover, the recording of this signal is possible thanks to the electrolyte fluxing that represents a significant added-value of this cell. In other conditions (such as bubble formation), the noise in the current signal won’t allow it. This aspect of course relates to point 1 of this review.
3. Interesting reviews have appeared on the topic and I think it might be worth citing them (for example: Deng *et al.*, 2020 ^2^).
4. Fig. 6 has no numbers at the x-axis, but the reader should be allowed to have an idea of the time scale of these experiments.

Is the rationale for developing the new method (or application) clearly explained?

Yes

Is the description of the method technically sound?

Yes

Are the conclusions about the method and its performance adequately supported by the findings presented in the article?

Yes

If any results are presented, are all the source data underlying the results available to ensure full reproducibility?

Yes

Are sufficient details provided to allow replication of the method development and its use by others?

Yes

Reviewer Expertise:

electrochemistry, photoelectrochemistry, X-ray spectroscopies

I confirm that I have read this submission and believe that I have an appropriate level of expertise to confirm that it is of an acceptable scientific standard, however I have significant reservations, as outlined above.

## References

[1] : Operando X-ray absorption spectroscopy of WO3 photoanodes. *Electrochimica Acta* .2019;320: 10.1016/j.electacta.2019.134561 10.1016/j.electacta.2019.134561

[2] : Understanding Photoelectrochemical Water Oxidation with X-ray Absorption Spectroscopy. *ACS Energy Letters* .2020;5(3) : 10.1021/acsenergylett.9b02757 975-993 10.1021/acsenergylett.9b02757

# Response

**Published:** 2022-11-30

**Authors:** Philipp Jäker

**Affiliations:** ETH Zürich, Switzerland

Dear Alessandro Minguzzi,

 We highly appreciate your encouraging and constructive report and would like to respond to the four comments you recommended us to consider.

1. We removed our claim that current cell versions are limited by electrochemical noise based on the following arguments/reasons
  1. Most publications do not provide adequate data/figures to quantitatively assess the signal-to-noise ratio (S/N) in electrochemical measurements, e.g. recordings of voltage or current. My claim was based on Figure S5 of Yoshida et al. ^1^ publication which lacks real physical units and an actual scale along the y-axis.
  2. We ourselves do not address the electrochemical S/N in the same manner we did to assess the S/N of the HERFD-XAS measurements although we claimed to do so. It only remains a qualitative, visual inspection of respective current and voltage measurements, which I do regard as insufficient given the S/N is a well-defined quantitative property. This would mean falling short regarding once own standards.
  3. However, a cell’s X‑ray beam attenuation on the basis of well-established Lambeert-Beer like exponential attenuation laws can be compared quite thoroughly once the energy range, absorption coefficient and material thickness are known for example using this web based application relying on tabulated values: https://henke.lbl.gov/optical_constants/.
    In our cell design the X-ray beam has to pass 500 nm of Si3N4 and 500 µm of approximately pure water, disregarding the minor acidic or basic electrolyte ion contribution.
    Please correct me if mistaken: Your 3D-printed cells described in your publication ^2^ I referenced were designed with a 100 µm Mylar or polyacrilic resin window followed by a 1 mm thick electrolyte layer which the X-ray beam had to pass.

The cell you referenced in your report ^3^ features a 10 µm Mylar window and an unclear electrolyte thickness, as you only provide two spatial dimensions (“2 x 2 mm²). Does one of those spatial dimensions describe the thickness along the X-ray beam axis, perpendicular to your working electrode surface and as such the electrolyte thickness?   Although, Si _3_N _4_ is about 2-3 times denser than the polymers you used the 20-200 times greater thickness is the defining parameter. In combination with at least a 2 times thicker electrolyte layer, the X-ray attenuation of our cell compared to your cells is significantly reduced.

 Additionally, your studies report XAS measurements at the L _III_ edges of W and Ir, slightly above 10 keV where attenuation becomes less severe as the energy dependent absorption coefficients generally decrease with increasing energy.

 Thus, I believe that “current cell versions often do not minimize X-ray beam attenuation” is an objectively fair statement to make based on combined empirical & modelled evidence.

1. I agree that we could have been more critical about the potential promises about X‑ray absorption measurements via ionic current detection. Therefore relevant text passages have been altered and a crucial recent publication has been referenced.

The work of van Spronsen et al. ^4^ illustrates how critical a deep understanding of electrical/electrochemical measurements is in order to decipher the current contributions upon X‑ray absorption. That is why we have not drawn any conclusions from our measured current other than that it clearly shows that X‑ray beam induced currents can be measured and that the current trace resembles X‑ray absorption Fe K-edge spectra measured *via* HERFD-XAS. However, I remain very optimistic that carefully analyzing this cell type’s measurement properties and possibly redesigning the cell might enable experiments to reveal more details about X‑ray beam induced currents and what information they can provide.

1. I appreciate your literature suggestions but without a clear statement which claims in our manuscript currently are not supported I refrain from citing it. I am aware of your suggested publication and I do not see crucially relevant information given they that has not already been covered in quite a number of recent reviews, even if the considered one focuses specifically on operando X‑ray absorption spectroscopy in photoelectrochemical systems. Additionally, in my view one should be careful in citing review articles instead of the relevant primary literature as this obscures a clear reference literature path and might even be used for gaming bibliometrics.
2. The HERFD-XAS scan duration was reported as 60 s in our experimental section. But agree that it is easier to read with appropriate axis labels and therefore added them to the figure, as well as the X‑ray on/off duration in the inset.

1.           Yoshida, M.; Yomogida, T.; Mineo, T.; Nitta, K.; Kato, K.; Masuda, T.; Nitani, H.; Abe, H.; Takakusagi, S.; Uruga, T.; Asakura, K.; Uosaki, K.; Kondoh, H., In situ observation of carrier transfer in the Mn-oxide/Nb:SrTiO3 photoelectrode by X-ray absorption spectroscopy. *Chem. Commun.* **2013,** 49, (71), 7848-7850.

 2.           Achilli, E.; Minguzzi, A.; Visibile, A.; Locatelli, C.; Vertova, A.; Naldoni, A.; Rondinini, S.; Auricchio, F.; Marconi, S.; Fracchia, M.; Ghigna, P., 3D-printed photo-spectroelectrochemical devices for in situ and in operando X-ray absorption spectroscopy investigation. *J. Synchrotron Rad.* **2016,** 23, (2), 622-628.

 3.           Fracchia, M.; Cristino, V.; Vertova, A.; Rondinini, S.; Caramori, S.; Ghigna, P.; Minguzzi, A., Operando X-ray absorption spectroscopy of WO3 photoanodes. *Electrochimica Acta* **2019,** 320, 134561.

 4.           van Spronsen, M. A.; Zhao, X.; Jaugstetter, M.; Escudero, C.; Duchoň, T.; Hunt, A.; Waluyo, I.; Yang, P.; Tschulik, K.; Salmeron, M. B., Interface Sensitivity in Electron/Ion Yield X-ray Absorption Spectroscopy: The TiO2–H2O Interface. *J. Phys. Chem. Lett* **2021,** 12, (41), 10212-10217.
